# Major QTLs Control Resistance to Rice Hoja Blanca Virus and Its Vector *Tagosodes orizicolus*

**DOI:** 10.1534/g3.113.009373

**Published:** 2013-11-15

**Authors:** Luz E. Romero, Ivan Lozano, Andrea Garavito, Silvio J. Carabali, Monica Triana, Natalia Villareal, Luis Reyes, Myriam C. Duque, César P. Martinez, Lee Calvert, Mathias Lorieux

**Affiliations:** * Rice Genetics and Genomics Laboratory, International Center for Tropical Agriculture (CIAT), A. A. 6713, Cali, Colombia; † Virology Unit, International Center for Tropical Agriculture (CIAT), A. A. 6713, Cali, Colombia; ‡ Rice Project, International Center for Tropical Agriculture (CIAT), A. A. 6713, Cali, Colombia; § DIADE Research Unit, Institut de Recherche pour le Développement (IRD), 34394 Montpellier Cedex 5, France

**Keywords:** rice (*Oryza sativa* L.), plant disease, tenuivirus, planthopper, molecular breeding

## Abstract

Rice hoja blanca (white leaf) disease can cause severe yield losses in rice in the Americas. The disease is caused by the rice hoja blanca virus (RHBV), which is transmitted by the planthopper vector *Tagosodes orizicolus*. Because classical breeding schemes for this disease rely on expensive, time-consuming screenings, there is a need for alternatives such as marker-aided selection. The varieties Fedearroz 2000 and Fedearroz 50, which are resistant to RHBV and to the feeding damage caused by *T. orizicolus*, were crossed with the susceptible line WC366 to produce segregating F_2:3_ populations. The F_3_ families were scored for their resistance level to RHBV and *T. orizicolus*. The F_2:3_ lines of both crosses were genotyped using microsatellite markers. One major QTL on the short arm of chromosome 4 was identified for resistance to RHBV in the two populations. Two major QTL on chromosomes 5 and 7 were identified for resistance to *T. orizicolus* in the Fd2000 × WC366 and Fd50 × WC366 crosses, respectively. This comparative study using two distinct rice populations allowed for a better understanding of how the resistance to RHBV and its vector are controlled genetically. Simple marker-aided breeding schemes based on QTL information can be designed to improve rice germplasm to reduce losses caused by this important disease.

Rice hoja blanca (white leaf) (RHB) disease has been reported in most countries that grown rice (*Oryza sativa* L.) in the Americas, including Peru, Ecuador, Colombia, Venezuela, Panama, Brazil, Belize, Puerto Rico, Costa Rica, Nicaragua, Honduras, El Salvador, Dominican Republic, Cuba, Guyana, Surinam, French Guyana, the United States, and Mexico (Garces-Orejuela *et al.* 1958; Morales and Jennings 2010; Morales and Niessen 1985). Epidemics of RHB occur sporadically, but with catastrophic results in terms of rice yields. Losses have been estimated at a country level to be as high as 25–50% of the crop (Jennings 1963; Vargas 1985). The symptoms in the rice plants are chlorotic streaks that can coalesce and cause the leaves to turn yellow or white. When young plants become infected they are stunted, and in severe infections the leaves turn necrotic and the plants die (Morales and Niessen 1983). Infections that occur before the emergence of the panicle can reduce seed set and grain quality. Moreover, it has been reported that RHB virus (RHBV) infection predisposes rice to *Helminthosporium oryzae* (Lamey and Everett 1967) and discoloration of the grain. There is indirect evidence that rice infected by RHBV may be more susceptible to other diseases.

The RHBV virus was isolated and partially characterized as a member of the *Tenuivirus* genus by Morales and Niessen (1983, 1985). It is closely related to other tenuiviruses that are found in the Americas, such as Echinochloa hoja blanca virus (de Miranda *et al.* 1996) and Urochloa hoja blanca virus (de Miranda *et al.* 2001). The type member of the tenuiviruses is rice stripe virus, another tenuivirus of rice, but it is not closely related to RHBV (Toriyama 1982). The tenuivirus found outside of the Americas that is most closely related to RHBV is Iranian wheat stripe virus (Heydarnejad and Izadpanah 1992; Calvert 2010). The molecular structure of the tenuiviruses has been described (Ramirez and Haenni 1994; Falk and Tsai 1998). Like other tenuiviruses, the RHBV is ambisense and RNA 2, 3, and 4 encode proteins on complementary RNA strands (Ramirez *et al.* 1992, 1993; Calvert *et al.* 1994). It has been shown that NS3 protein of RHBV can suppress RNA silencing in rice and *Tagosodes orizicolus* by binding both siRNAs and miRNAs (Hemmes *et al.* 2007, 2009). Transgenic rice that was developed as an alternative source of resistance to RHBV is believed to bring resistance through gene silencing (Lentini *et al.* 2003). It is suspected that plant resistance may involve mechanisms to overcome the ability of the NS3 protein to effectively suppress gene silencing.

The planthopper *Tagosodes orizicolus* Muir (Homoptera: Delphacidae, formerly known as Sogata orizicola, Sogatodes orizicola, or Sogatodes oryzicola, and commonly called “sogata” in Latin America) is both a host and vector of RHBV (Galvez *et al.* 1961). After the virus is acquired through feeding on infected plants, there is a period of 17–22 days of viral propagation before the planthopper becomes viruliferous (Galvez 1968; Webber *et al.* 1971). There is a high rate of *trans*-ovarian transmission to the progeny, and the nymphs can transmit the virus soon after they emerge. Resistance to RHBV is found in populations of *T. orizicolus*, and a high proportion of insects are immune to virus infection (Zeigler and Morales 1990). It was estimated that during outbreaks of RHB 15–25% of the planthoppers were able to transmit (potential vectors) the virus (Galvez 1968; Reyes *et al.* 1997). During outbreaks of RHB, most of the potential vectors become infected with RHBV. The low fecundity of the RHBV-infected planthoppers and genetic resistance are the probable principal factors for the cyclic nature of the RHB epidemics (Zeigler and Morales 1990). Apparently, in the absence of selective pressure for resistance, the percentage of the genetic ability of the insect to transmit the virus tends to increase.

Researchers have been trying unsuccessfully to mechanically transmit the virus to rice (L. E. Romero *et al.*, unpublished data; F. Morales, personal communication). Viruliferous insect colonies that are produced by the crossing of proven *T. orizicolus* vectors are used to inoculate the plants and select RHBV-resistant varieties (Zeigler *et al.* 1988). Maintaining viruliferous colonies for a breeding program requires a costly insect growing and breeding effort. Field screening in a typical breeding program for RHBV resistance is a two-step process. In the early generations, the segregating populations are tested using moderate vector pressure (*i.e.*, average of one to two insects per plant). This step helps to discard the most susceptible lines but does not guarantee that the subsequent progeny will harbor good resistance levels. After additional selection in segregating generations and fixation of the traits by selfing, the materials are tested using plants of different ages and higher vector pressure (*i.e.*, average of three to four insects per plant). More precise screening can be performed under greenhouse conditions in limited population sizes, in which the number of vectors per plant can be controlled and feeding on all genotypes can be forced. Greenhouse trials generally provide more repeatable and precise results than field trials.

Plant response to RHBV depends on plant age at inoculation and the concentration of RHBV inoculate. To date, all varieties that have been tested could be infected as long as the plants are young enough and the virus pressure is sufficiently high. The level of resistance of a genotype to RHBV is determined by the percentage of plants that become infected. A screening for RHBV relies on a complex biological system that involves vector colonies that can vary in the percentage of vectors (*i.e.*, of infected insects) and the concentration of the virus in the vectors. Moreover, environmental conditions (*e.g.*, temperature, solar radiation, plant density, leaf color, tillering, and others) can affect the feeding behavior of the insects. This makes it difficult to ensure optimum levels of infection by RHBV. Because of the technical difficulties and associated costs, few rice breeding programs use viruliferous colonies of *T. orizicolus* to develop RHBV-resistant varieties. Consequently, most of the resistant cultivars have been developed through screening at the International Center for Tropical Agriculture (CIAT) at Palmira headquarters, Colombia. Therefore, it is important to develop efficient technologies that are cost-effective and time-effective for breeding this trait. One promising approach is selection based on molecular markers linked to resistance genes. The aim of this investigation was to contribute to a better understanding of the genetics of RHB resistance through the identification of QTL for resistance to RHBV and to its vector *T. orizicolus*.

## Materials and Methods

### Genetic materials

Using parents with contrasting levels of resistance to RHBV and *T. orizicolus*, two intraspecific *O. sativa* ssp. indica × *O. sativa* ssp. japonica populations suitable for QTL analysis and segregating for resistance to RHBV and *T. orizicolus* were developed. The first population consisted of F_2:3_ lines derived from the F_1_ between Fedearroz 2000 (Fd2000; CIAT CT10323-29-4-1-1T-20), an indica cultivar from Colombia, and the tropical japonica accession WC 366 (IR65598-27-3-1). Fd2000 is highly resistant to RHBV and moderately resistant to *T. orizicolus*. WC 366 is highly susceptible to RHBV and *T. orizicolus*. F_3_ seeds were obtained from bagged panicles of 218 F_2_ plants. The second population was developed in a similar way and consisted in 291 F_2:3_ lines derived from the F_1_ between Fedearroz 50 (or Fd50), an indica cultivar from Colombia, and WC 366. Fd50 is highly resistant to feeding damage caused by the insect vector and shows intermediate resistance to RHBV.

### Evaluation for resistance to RHBV and *T. orizicolus*

The F_3_ families, their parents, and control lines were evaluated for resistance to RHBV and feeding damage caused by the vector insect in the greenhouse facilities at the CIAT headquarters in Palmira, Colombia. The F_3_ plants were evaluated in trays filled with sterilized soil. For each F_3_ family, three rows of 20 plants each were used. Rows were distributed randomly in the trays. The two crosses were evaluated in separate experiments, and each experiment was performed twice.

#### Resistance to the virus:

Plants were infested at 15 days after sowing with a dosage of 1.5 insects per plant using insects from the colony “Costa-CIAT,” which was developed using viruliferous insects collected from Monteria (Northern Colombia). Between 70% and 90% of the insects were virulent for RHBV. The average temperature was 27° and average relative atmospheric humidity was 80%. Five days after infestation, the insects were eliminated using an insecticide, and the plants were evaluated for symptoms of RHBV at 14, 21, and 28 d after inoculation. The following controls were randomly placed in the trays: Colombia 1 (resistant); Oryzica 1 (intermediate); and Bluebonnet 50 (susceptible). In each experiment, the percentage of plants showing virus symptoms in each F_3_ family, or percentage of incidence index (PII), was calculated from the 60 plants evaluated, and the mean value of the two experiments was used as a measure of the level of susceptibility to RHBV.

#### Resistance to the insect:

Feeding damage generated by the nymphs and adults of *T. orizicolus*, which feed on the mesophyll and phloem, produces accelerated senescence, yellowing, and necrosis of leaves from the apex and edges toward the basal part of the plant. It slows plant growth and can cause plant death. Generally, females cause more severe damage than males do, because of feeding and oviposition (Zeigler *et al.* 1994). Plants were infested at 15 days after sowing, with approximately 10 nonviruliferous nymphs of *T. orizicolus*, and the insects were allowed to feed on the plants until the death of at least 85% of the plants in the highly susceptible control Bluebonnet 50. The controls, Bluebonnet 50, Makalioka (resistant), and Cica 8 (intermediate), were randomly placed in the trays. In each experiment, the percentage of dead plants (PDP) in each F_3_ family was calculated from the 60 plants evaluated, and the mean value of the two experiments was used as a measure of the level of feeding damage caused by *T. orizicolus*.

### Plant DNA isolation

Leaf tissue was collected from 15-day-old F_2_ plants or bulks of 15 plants per F_3_ family. Samples were frozen in liquid nitrogen and stored at −80° until processed. Plant DNA was isolated in plates containing 96 wells with a volume of 1.2 ml, using a modified version of the CTAB method (Murray and Thompson 1980) as follows: 480 µl extraction buffer was added to 150 mg ground frozen leaf tissue. The buffer was 100 mM Tris (pH = 8.0), 1.4 M NaCl, 20 mM EDTA (pH = 8.0), MATAB 2%, sodium bisulfite 0.5%, and PEG 8000 1%. This mixture was incubated in a water bath at 74° for 30 min. Subsequently, 480 µl chloroform:isoamyl-alcohol (24:1) was added and the mixture was centrifuged at 11,000 rpm. Supernatants were precipitated with 250 µl isopropanol at −20° for 15 min and centrifuged at 11,000 rpm. The pellets were washed with 250 µl 70% ethanol and allowed to dry by inverting the tubes for 30 min. DNA was resuspended in Tris-EDTA [10 mM Tris-HCl (pH = 8.0) and 1 mM EDTA (pH = 8.0)] and was quantified using a Hoefer DyNA QUANT 200 fluorometer.

### Parental DNA polymorphism tests

A set of 173 simple-sequence repeat (SSR) DNA markers was evaluated in the parents Fedearroz 2000, Fedearroz 50, and WC366 of the crosses. The SSRs were selected from the Gramene database (http://www.gramene.org) for their even distribution across the rice genome (McCouch *et al.* 2002). Polymerase chain reactions (PCRs) were performed using commercial primers from ResGen (Life Technologies Corporation) and IDT (Integrated DNA Technologies). Optimal PCR conditions were determined by varying the magnesium concentrations and the binding temperature. Briefly, the PCRs were performed in a final volume of 15 μl using 20 ng/µl DNA, 10 M primers (forward and reverse), 10 mM dNTPs, 1.5 to 2.5 mM MgCl_2_, 1× PCR buffer, Taq polymerase, and sterile water.

Subsequently, denatured samples were loaded onto 4% polyacrylamide gels (29:1 acrylamide:bisacrylamide) containing 5 M urea and 0.5× TBE and run at 90 W at 50°. A 10-bp DNA ladder was used to estimate the size of the alleles. Gels were stained with silver nitrate.

### Genotyping of F_2:3_ populations

The polymorphic SSR markers were used to evaluate the F_2_ or the bulked F_3_ families when F_2_ DNA was scarce. Polymorphisms smaller than 10 bp were run on 4% polyacrylamide gels and stained with silver nitrate as described. Polymorphisms larger than 10 bp were run on 4% agarose gels and stained with SYBER-Safe (Life Technologies Corporation).

First, a bulked-segregant analysis (BSA) was performed using 30 resistant and 30 susceptible lines of each cross. Phenotypic values used to choose extreme lines were based on the final scoring for both RHBV and feeding damage of *T. orizicolus*. The lines were scored using 113 SSRs in the cross Fd2000 × WC366 and 65 SSRs in the cross Fd50 × WC366. Subsequently, the putative QTL regions identified by the BSA approach were saturated using additional SSRs evaluated in the entire populations, that is, 218 lines in Fd2000 × WC366 and 291 lines in Fd50 × WC366 crosses. Both genotyping and phenotyping data are available as two Qgene data files (.qdf; links to download the data are provided in Supporting Information, File S1).

### Statistical analysis and QTL mapping

Goodness-of-fit to Mendelian segregation (1:2:1) was tested by computing the chi-squared (χ^2^) statistic for each marker using MapDisto version 1.8.1 (Lorieux 2012; http://mapdisto.free.fr). The linkage map was computed using the same program. Marker order was determined using the order, ripple, and check inversions functions and were compared to the order on the physical map of the *O. sativa* genome (MSU version 7.0, http://rice.plantbiology.msu.edu). Markers showing incongruent positions between the genetic and the physical maps were removed from the analysis. Genotyping errors were detected and corrected using the iterative error detection function, running 15 iterations (start = 0.0001; step = 0.0001). Missing data were inferred using the replace missing data by flanking genotypes function, with a maximum probability of double recombination set to 0.001. Genetic distances of the final map were reported in centimorgans and estimated with the Kosambi mapping function (Kosambi 1944).

Analyses of distribution of the phenotypic traits as well as QTL detection were performed using the Qgene version 4 program (Nelson 2005; http://www.qgene.org). Data files were prepared using the export map and data function of MapDisto. For QTL detection, the following different methods were compared: single-marker regression; simple interval mapping; and composite interval mapping. The forward cofactor selection option was used in composite interval mapping. The LOD score statistic was used for all methods to make the results comparable. Empirical thresholds to declare presence of a QTL were obtained using the resampling by permutation method, performing 1000 iterations for each trait/chromosome combination. A QTL was declared positive if it was detected by all the three methods. Moreover, to correct for possible erroneous phenotypic data corresponding to escape, mis-scoring, or incomplete penetrance, all positive QTL were additionally confirmed by analysis of outliers in the trait distribution using the plot trait *vs.* genotype module of MapDisto. This module allows calculating corrected single-marker regression F-test values after detecting and removing outlier data in each marker genotypic class.

In the case of a secondary LOD score peak linked to a major peak of a QTL, to determine if the secondary peak corresponded to a true QTL or to an artifact—or “ghost QTL”—a detailed analysis of the distribution of recombination fractions along the chromosome was performed. The analysis looked for restriction of recombination fractions that could induce artificial linkage disequilibrium between the major and the secondary LOD score peaks. If artificial linkage disequilibrium was detected, then the secondary peak was declared an artifact.

## Results and Discussion

### RHBV symptoms

**Figure 1:**
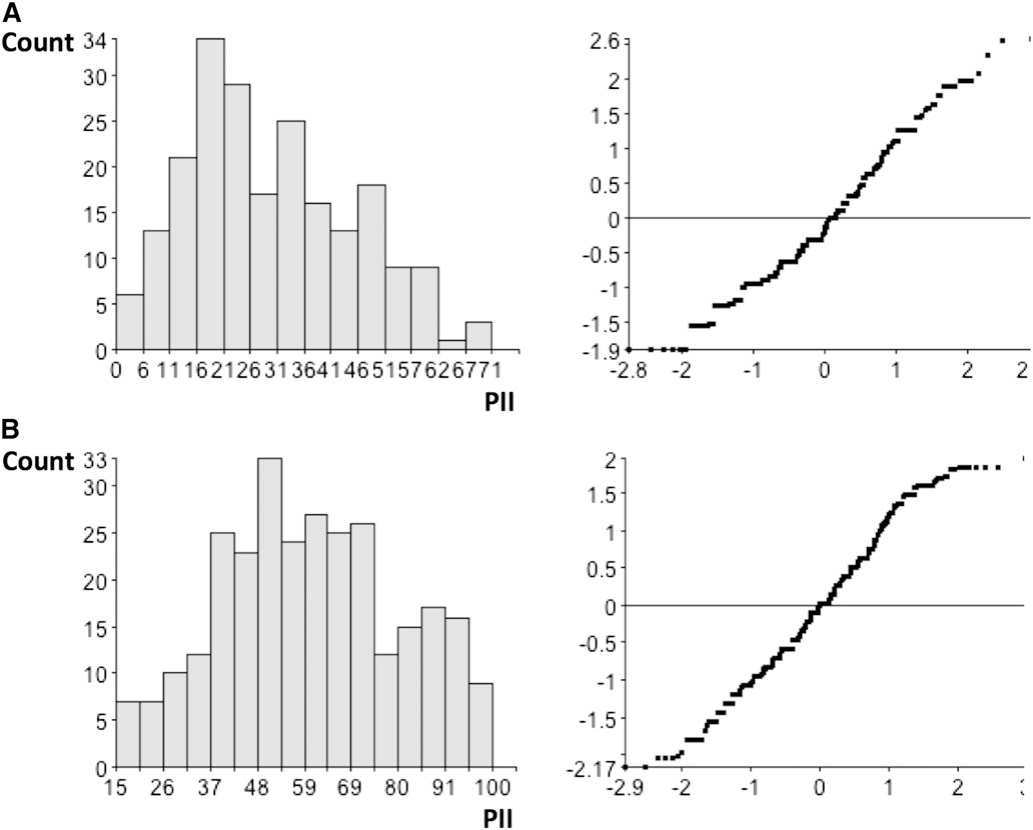
Trait distribution (left) of virus symptoms expressed as percentage of incidence index (PII) and associated normal score plot (right). (A) Fd2000 × WC366 cross. (B) Fd50 × WC366 cross.

In the Fd2000 × WC366 experiment, the parental lines and controls displayed the following PII scores: Fd2000, 4.5%; Oryzica 1, 16.8%; and WC366, 58.7%. In the F_3_ families, PII scores for RHBV symptoms ranged between 0% and 70.6% (see distribution in Figure 1A). The relatively low PII scores for the susceptible WC366 parent and the intermediate control Oryzica 1 and the maximum PII score significantly lower than 100% in the F_3_ lines indicate good, but not optimal, inoculation/infection efficiency. This may slightly lower the power of QTL detection and may lead to slightly underestimating the effects of QTL for RHBV resistance in this experiment.

In the Fd50 × WC366 experiment, the parental lines and controls displayed the following PII scores: Fd50, 27.1%; Fd2000, 3.2%; Bluebonnet 50, 87.1%; and WC366, 81.8%. In the F_3_ families, PII scores for RHBV symptoms ranged between 15.0% and 100.0% (Figure 1B). These scores are consistent with previous observations on the parental and check lines. Together with the distribution of PII scores in the F_3_ population, the data indicate very good inoculation/infection efficiency in the experiment.

### *T. orizicolus*–feeding damage symptoms

In the two experiments, symptoms of feeding damage attributable to feeding by *T. orizicolus* confirmed the following previous observations: the resistant control Makalioka and the Fd50 parent exhibited PDP <10%; the moderately resistant parent Fd2000 showed a PDP of approximately 20%; the intermediate resistant cultivar Cica 8 showed a PDP of approximately 40%; and the susceptible parent WC366 and the susceptible control Bluebonnet 50 showed a PDP >85%.

**Figure 2:**
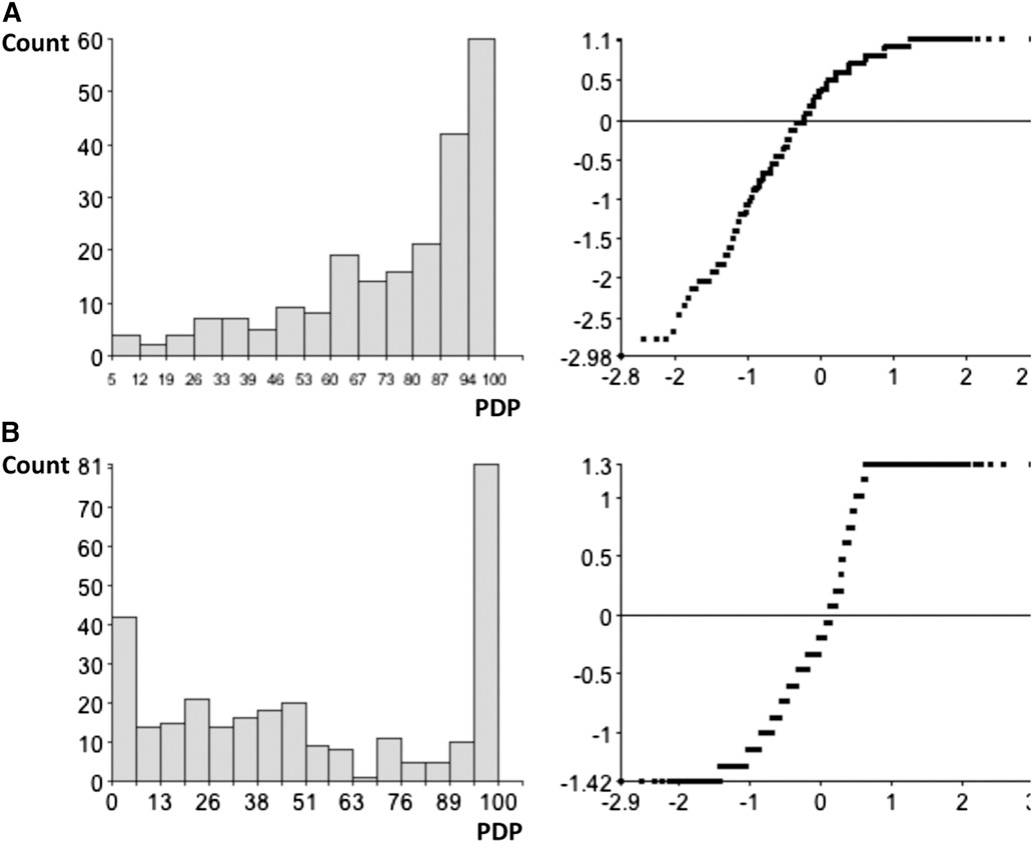
Trait distribution (left) of planthopper-induced feeding damage expressed as percentage of dead plants (PDP) and associated normal score plot (right). (A) Fd2000 × WC366 cross. (B) Fd50 × WC366 cross.

In the Fd2000 × WC366 experiment, the PDP scores of the F_3_ families ranged between 5% and 100.0%, with few families exhibiting very high resistance (see distribution in Figure 2A).

In the Fd50 × WC366 experiment, the PDP scores of the F_3_ families ranged between 0% and 100.0% (Figure 2B). A significant proportion (31.2%) of the population showed high or very high resistance (PDP <10%) to *T. orizicolus*, suggesting that a major resistance gene controls the resistance in Fd50.

### One major QTL for RHBV resistance

**Figure 3:**
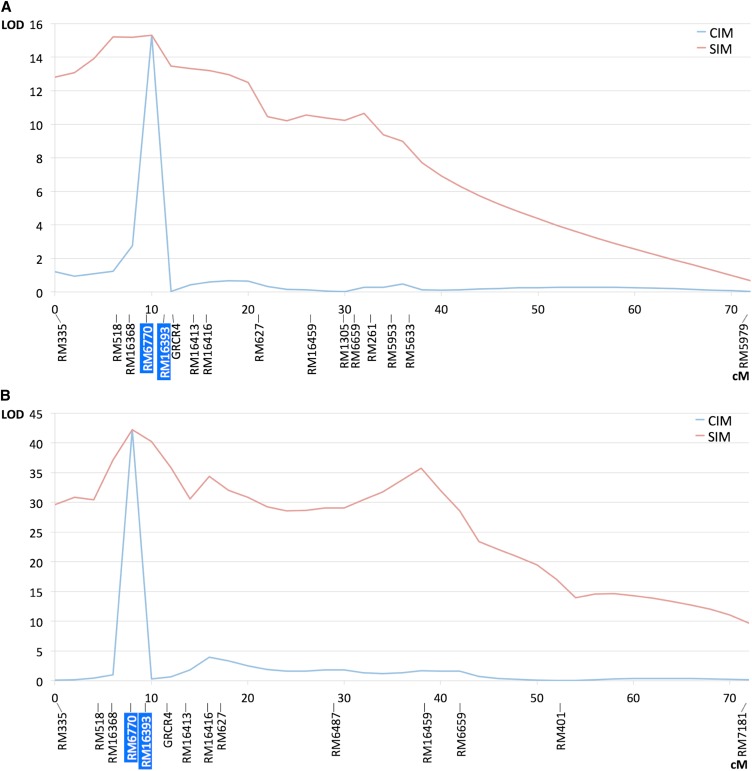
LOD score plot for resistance to RHBV in two F_2:3_ populations on rice chromosome 4. (A) Fd2000 × WC366 cross (*P* = 0.01; permutation threshold, 4.11). (B) Fd50 × WC366 cross (*P* = 0.01; permutation threshold, 3.34). CIM, composite interval mapping; SIM, simple interval mapping. Markers highlighted in blue were selected as cofactors.

In both crosses, the BSA approach allowed us to associate one region of the short arm of chromosome 4 with RHBV resistance. Genotyping of the entire populations with more SSR markers in the chromosomal region allowed us to determine the QTL position in the two populations. As shown in Figure 3, A and B, the composite interval mapping analysis indicated very similar positions for the two QTL close to marker RM6770, indicating that the QTL corresponds either to the same gene or to two closely linked genes in Fd2000 and Fd50. The LOD scores and percentages of variance explained by the QTL were LOD = 15.3 and *R*^2^ = 0.28 in the Fd2000 × WC366 cross, and LOD = 42.2 and *R*^2^ = 0.49 in the Fd50 × WC366 cross (Table 1). These highly significant statistics indicate that RHBV resistance is mainly of monogenic control in both Fd2000 and Fd50. In Fd50, this QTL is probably the only one that contributes significantly to the trait, because one would barely observe *R*^2^ > 0.5 given the low precision of the PII scoring attributable to the erratic feeding behavior of the planthopper (Table S1). In Fd2000, the LOD score and *R*^2^ values were smaller than in Fd50 (Table S2), which could indicate a possible additional contribution of undetected QTL of smaller effect. However, this is more likely an effect of the lower infection/inoculation efficiency in our experiment, as explained (see *RHBV symptoms*), and because the QTL also corresponds to a major gene. The fact that no other QTL was detected in this cross also supports the hypothesis of a single, major QTL. In the two crosses, heterozygous individuals at marker RM6770 showed intermediate PII scores compared to the homozygotes, indicating that the two QTL effects are mostly of the additive type (Table 1).

**Table 1:** Summary of QTL found for RHBV and *T. orizicolus* resistance in two indica × tropical japonica crosses

Trait	Cross	Chromosome	Markers	Pos, Mbp	LOD score	*R*^2^	Additivity	Dominance
RHBV	Fd2000 × WC366	4	RM6670	2.81	15.3	0.28	−13.11	−0.90
			RM16393	3.41				
RHBV	Fd50 × WC366	4	RM6670	2.81	42.2	0.49	−21.19	−4.78
			RM16393	3.41				
*T. orizicolus*	Fd2000 × WC366	5	RM13	2.89	14.4	0.26	−16.14	11.09
			RM17962	3.85				
*T. orizicolus*	Fd50 × WC366	7	RM560	19.58	43.3	0.50	−38.91	−9.04
			RM346	21.04				

Pos, position on MSU 7.0 reference rice genome in megabase pairs (Mbp).

### Two QTL for *T. orizicolus* resistance

For resistance to *T. orizicolus*, the BSA approach allowed the identification of one region of the short arm of chromosome 5 in the cross Fd2000 × WC366 and one region of the long arm of chromosome 7 in the cross Fd50 × WC366.

**Figure 4:**
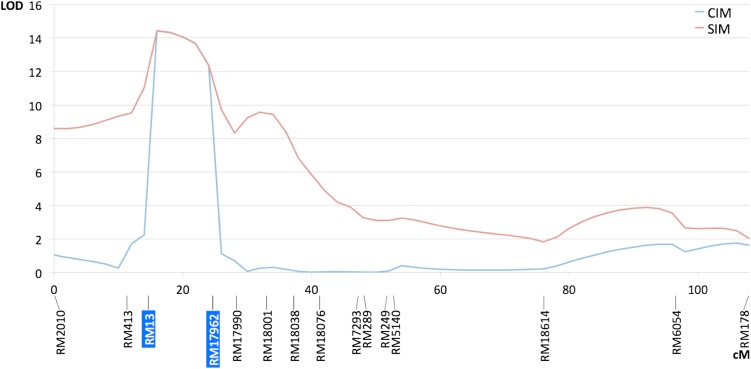
LOD score plot for resistance to *T. orizicolus* on rice chromosome 5 in the F_2:3_ population Fd2000 × WC366 (*P* = 0.01; permutation threshold, 4.48). CIM, composite interval mapping ; SIM, simple interval mapping. Markers highlighted in blue were selected as cofactors.

Additional genotyping of the entire Fd2000 × WC366 population led to the mapping of a major QTL located between the markers RM13 and RM17962 on chromosome 5 (Figure 4), with LOD = 14.4 and *R*^2^ = 0.26 (Table 1 and Table S3). No other QTL was found for this trait in this cross, indicating a mostly monogenic control of resistance to the planthopper in Fd2000. The QTL effect was mostly of the dominant type (Table 1), with the mean of heterozygotes being very similar to mean of the WC366 homozygotes.

**Figure 5:**
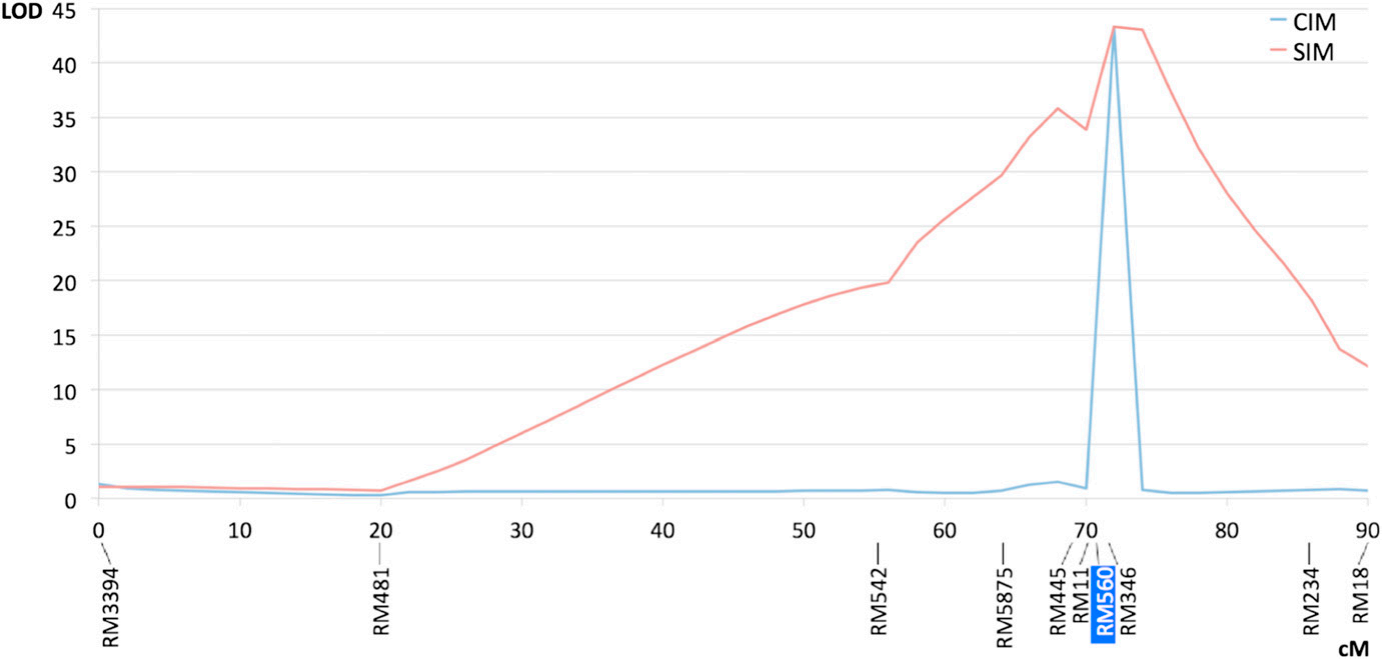
LOD score plot for resistance to *T. orizicolus* on rice chromosome 7 in the F_2:3_ population Fd50 × WC366 (*P* = 0.01; permutation threshold, 3.22). CIM, composite interval mapping; SIM, simple interval mapping.. Markers highlighted in blue were selected as cofactors.

Similarly, additional genotyping of the entire Fd50 × WC366 population led to the mapping of a major QTL close to RM560 on chromosome 7 (Figure 5), with LOD = 43.3 and *R*^2^ = 0.50 (Table 1 and Table S4). The high values of the statistics strongly support the hypothesis of monogenic control of resistance to the planthopper in Fd50. The QTL effect was mostly of the additive type (Table 1).

### Independent control of *T. orizicolus* resistance QTL and RHBV resistance

Because the RHBV is transmitted by *T. orizicolus*, the hypothesis that *T. orizicolus* resistance can alter the response to RHBV was tested. The two traits were not correlated in either of the two populations (*R*^2^ = 0.003 in Fd2000 × WC366; *R*^2^ = 0.08 in Fd50 × WC366). Moreover, no significant LOD score for RHBV resistance was found at the QTL positions on chromosomes 5 and 7 for feeding damage. Furthermore, classifying the lines according to their allelic configuration at the two QTL for RHBV and *T. orizicolus* resistance shows that the lines that have the Fd2000 allele at the two QTL and the lines that have the Fd2000 allele at the RHBV resistance QTL and the WC366 allele at the *T. orizicolus* resistance QTL show similar resistance to RHBV. This is also true for the subpopulations that have the Fd50 allele at the two resistance QTL on chromosomes 4 and 7 (data not shown). These results indicate that the two traits are controlled mostly by independent genetic factors.

### Segregation distortion in RHBV resistance QTL region

On chromosome 4, where a strong QTL for RHBV resistance was found, complex patterns of segregation distortion were observed in the two crosses, with a maximum χ^2^ for deviation from Mendelian 1:2:1 expectations found at the same location (marker RM16413). In the Fd50 × WC366 cross, neither of the parental alleles was favored at RM16413, but the heterozygotes were favored over the homozygotes. In the cross Fd2000 × WC366, the heterozygotes were favored, too; however, the Fd2000 allele was slightly favored over the WC366 allele (Table S5 and Table S6). We show that these segregation patterns are likely to alter the shape of the LOD score statistic curve along chromosome 4 in the two crosses. Segregation patterns followed Mendelian 1:2:1 expectations at the QTL locations for both feeding damage on chromosome 5 in the cross Fd2000 × WC366 and chromosome 7 in the cross Fd50 × WC366.

Segregation distortion is commonly observed in intersubspecific *O. sativa* ssp. indica × *O. sativa* ssp. japonica crosses (Harushima *et al.* 1996; Xu *et al.* 1997; Wu *et al.* 2010; Ouyang and Zhang 2013). It is usually attributable to the effect of sterility genes that induce gametic selection, which is probably the case in our study. Other studies report intraspecific segregation distortion in the region of the QTL of RHBV resistance on the short arm of chromosome 4 (Xu *et al.* 1997; Harushima *et al.* 2001; Wu *et al.* 2010). This phenomenon generally does not modify or slightly modifies QTL detection, because allelic disequilibrium does not affect the genotypic means of a given trait. However, complex patterns of segregation distortion that involve several linked sterility genes, or extreme deviations from Mendelian proportions, may significantly alter estimation of recombination fractions, map orders (Lorieux *et al.* 1995; Liu *et al.* 2010; Zhan and Xu 2011) and QTL detection (Xu 2008; Zhang *et al.* 2010). The case of the QTL region on chromosome 4 in our data illustrates the importance of looking carefully at the recombination fraction distribution and the segregation distortion pattern within and around the QTL regions.

### Ghost QTL for RHBV resistance?

**Figure 6:**
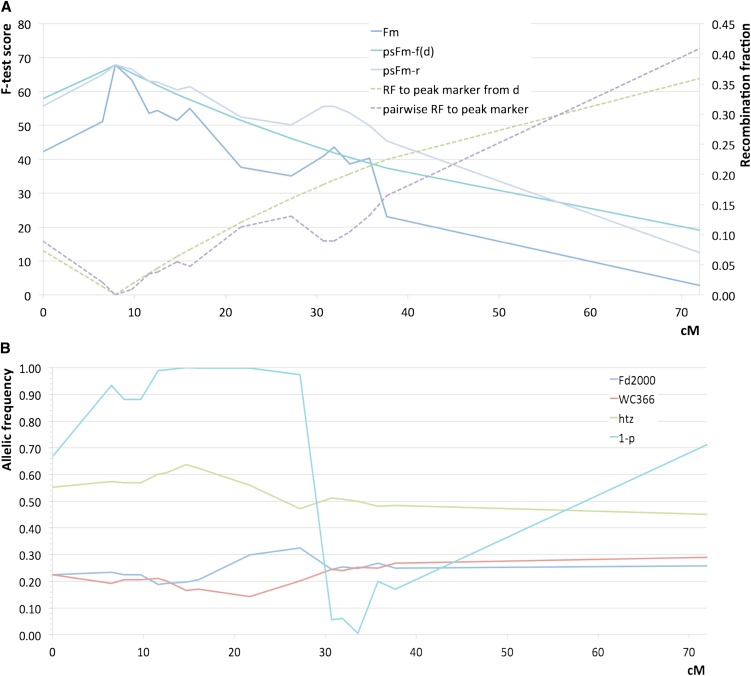
(A) Comparison of single-marker F-test and pseudo F-tests for RHBV resistance along chromosome 4 in the F_2:3_ population Fd2000 × WC366. F-test at marker *m* (*F_m_*) is calculated by single-marker regression analysis. Pseudo F-tests are defined as ps*F_m_* = *F_peak_*(1 − 2*r*), where *F_peak_* = *F_m_* at the closest marker to the QTL peak (or “peak marker”) and *r* is either the observed pairwise recombination between marker *m* and the peak marker (ps*F_m_* −*r*) or the recombination fraction *r*(*d*) obtained by inverting the Haldane mapping function of the sum of the adjacent interval sizes *d* (in cM) that separate marker *m* and the peak marker [ps*F_m_−f*(*d*)]. Similarity of *F_m_* and ps*F_m_* −*r* curve shapes, which both show a secondary peak at marker RM6659 (position 52.7 cM), strongly indicates that this peak is not attributable to another QTL at RM6659 but to restriction of recombination between the true peak marker RM16368 and marker RM6659, which artificially increased the *F_m_* value at marker RM6659. This is confirmed by the absence of secondary peak in the ps*F_m_ −f*(*d*) curve, which does not depend on recombination restriction. Comparison of pairwise recombination fraction and *r*(*d*) between marker *m* and the peak marker along chromosome 4, evidencing the restriction of recombination between marker RM6659 and the peak marker. (B) Allelic frequencies of the three genotypes (homozygous Fd2000, htz = heterozygote and homozygous WC366) at marker *m* and 1 − *P* values, where *P* is the probability associated with the segregation χ^2^ statistic, along chromosome 4. Comparison of (A) and (B) curve shapes tends to indicate that restriction of recombination between marker RM6659 and the peak marker RM16368 is probably attributable to the strong segregation distortion pattern between these loci, with values of 1 − *P* close to 1 in this region.

A detailed analysis of recombination fractions and deviation from expected segregation indicates that the secondary QTL peaks on chromosome 4 for RHBV resistance observed at marker RM6659 (Fd2000 × WC366 cross) and marker RM16459 (Fd50 × WC366 cross) are probably artifacts, or “ghost QTLs.” This is because of the restricted recombination between their respective markers and RM6770, which is the marker most strongly associated with RHBV resistance (Figure 6A). This restriction of recombination is likely to be attributable to the strong segregation distortion between these loci, as explained in detail in Figure 6B (Lorieux *et al.* 1995).

### On the QTLs found

This study represents the first report of resistance QTL for RHB disease and *T. orizicolus*, both of which can cause severe losses in rice crops in the Americas. We found three important QTL in the two resistant cultivars Fedearroz 2000 and Fedearroz 50: one QTL or two tightly linked QTL for RHBV resistance, and two QTL for resistance to *T. orizicolus* (Table 1, Table S1, Table S2, Table S3, and Table S4). These results have important implications for rice breeding in Latin America because they open the way for the use of modern, efficient breeding strategies to obtain new elite germplasm with high resistance to RHB disease.

All the QTL were supported by very strong statistical significance, with LOD scores ranging between 14.4 and 43.4, and strong *R*^2^, which clearly classify them as major QTL. The statistics were even more significant in the Fd50 × WC366 cross than in the Fd2000 × WC366 cross. This is likely to be attributable to better inoculation/infection efficiency in the Fd50 × WC366 experiment. Moreover, the population size, which also contributes to increased significance, was larger in the Fd50 × WC366 cross (291 F_3_ lines) than in the Fd2000 × WC366 cross (218 F_3_ lines).

The mapping resolution of the QTL for RHBV resistance on chromosome 4 did not allow us to tell if they actually correspond to the same QTL in both crosses or to distinct, tightly linked genes. We plan to perform fine-mapping experiments for this QTL region and expect to be able to answer this pending question in the near future.

### What the QTL tells us about the RHBV resistance mechanism

Although knowing the number and individual effects of QTL underlying a trait does not provide direct information regarding its biological or molecular mechanisms, it can help interpret other data. It was found recently that the RHBV has developed a strategy of binding siRNAs through its NS3 protein to suppress the RNA interference strategy used by the plant against the virus (Goldbach *et al.* 2003; Hemmes *et al.* 2007, 2009; Yang *et al.* 2011). Deploying viral suppressors of RNA silencing is a common strategy that viruses use to multiply in plants (Lakatos *et al.* 2006; Nakahara *et al.* 2012; Sansregret *et al.* 2013). Because binding efficiency directly depends on molecule concentration in the cell (dosage effect), heterozygous plants carrying a single copy of a resistance allele that interferes with the binding process could show intermediate phenotype if the QTL actually alters the molecular interaction mechanism. In the case of RHBV resistance, the QTL on chromosome 4 is mainly additive, meaning that the heterozygotes have intermediate values of resistance. Thus, our data are compatible with the hypothesis that this QTL alters the molecular interaction mechanism described by Hemmes *et al.* (2009) in hampering the binding process, for instance, in blocking the loading of siRNAs into RNA silencing effector complexes. Nevertheless, more data are needed to verify this hypothesis and the possibility of other defense mechanisms cannot be rejected.

### Usefulness of QTL for breeding

RHB disease is a situation in which marker-aided breeding has a great advantage over classical selection because being able to select individuals that bear favorable gene or QTL alleles for RHBV and *T. orizicolus* resistance would eliminate the need for expensive, poorly reliable, and time-consuming field screening trials of selfed families during the selection scheme. It would also be much more accurate and, additionally, would allow for better control of foreground and background genomes, reducing the number of generations required to recover the recipient genotype (Hospital 2001). This would also allow any breeding program lacking facilities to perform field or greenhouse screening to select for RHB resistance, provided that they have access to simple molecular marker technology or to outsourced genotyping services. For varieties that will be released as resistant to RHBV and *T. orizicolus*, it is advisable to confirm both the planthopper and virus resistance using the appropriate biological system.

We found that the resistance to both RHBV and the planthopper vector is controlled by major QTL. Thus, marker-aided selection schemes based on these QTL are easy to design and implement and require easily manageable population sizes at each generation. A pilot marker-assisted backcross selection program was initiated in collaboration with the Colombian national breeding program Fedearroz, which comprised the introgression of the Fd2000 and Fd50 QTL in two elite lines adapted to Colombian growing conditions. BC_3_F_4_ lines were selected on the sole criteria of the QTL and genetic background SSR markers, with no phenotyping at any step. The lines were then evaluated during the second semester of 2012 in the greenhouse at CIAT-Palmira and on an RHB hotspot at Fedearroz experimental station in Cúcuta, Norte de Santander, Colombia. In both experiments, the BC_3_F_4_ lines showed significantly higher resistance to RHBV and *T. orizicolus* (L. E. Romero *et al.*, our unpublished data) compared to the recipient elite lines. Based on these encouraging results, the CIAT-Fedearrroz research team decided to extend the marker-assisted backcross selection program to four new genetic backgrounds to cover all the rice-growing environments in Colombia. We chose to use the two QTL from Fd2000 because of the much stronger resistance level of Fd2000 to RHBV compared to Fd50. However, we also plan to combine the Fd2000 QTL for RHBV and planthopper resistance with the Fd50 QTL for planthopper resistance in future breeding campaigns. Other national programs are encouraged to start similar breeding programs to improve their local genetic stocks for RHB resistance.

One may question the usefulness of planthopper resistance QTL, because the RHBV resistance QTL seems to act genetically independently and is sufficient to bring high levels of resistance to the virus under controlled greenhouse or field conditions in which viruliferous vectors are placed on the plants to be tested. Nevertheless, it is also important to regulate the insect populations to avoid epidemic bursts (Morales and Jennings 2010). For instance, large-scale cultivation of Fd50, which had strong antibiosis against *T. orizicolus*, helped in controlling RHB disease for many years in Colombia. Additionally, resistance to the planthopper can significantly reduce the yield losses attributable to the feeding damage. The planthopper is a major rice pest and varieties that lack good resistance require frequent applications of pesticides or suffer major losses from the pest. Furthermore, there is evidence that suggests that to have good field resistance to RHBV, the variety needs to have both resistance to the virus and its vector (Morales and Jennings 2010). The variety Llanos 5 has resistance to RHBV but is susceptible to *T. orizicolus*. In RHB outbreaks in the mid 1990s, fields of Llanos 5 often had higher percentages of plants infected than susceptible varieties with resistance only to the planthopper. This suggests that planthopper resistance is needed for RHB resistance in field conditions.

## Supplementary Material

Supporting Information
