# Supplementary material for: Major QTLs Control Resistance to Rice Hoja Blanca Virus and Its Vector Tagosodes orizicolus
Source: G3 (Bethesda). 2013 Nov 15;4(1):133–42. doi: 10.1534/g3.113.009373 (PMC3887529; doi:10.1534/g3.113.009373)
Supplement: Supporting Information [file supp_4_1_133__index.html]

Major QTLs Control Resistance to Rice Hoja Blanca Virus and Its Vector Tagosodes orizicolus — Supporting Information 

# Major QTLs Control Resistance to Rice Hoja Blanca Virus and Its Vector *Tagosodes orizicolus*

## Supporting Information for Romero *et al.*, 2014

**Files in this Data Supplement:**

- Supporting Information - File S1 and Tables S1-S6 (PDF, 446 KB)
- Table S5 - Genotypic classes sizes at SSR markers along chromosome 4, and chi-squared (*χ*2) statistic for goodness-of-fit with Mendelian 1:2:1 expectations, in the cross Fd2000 x WC366. (PDF, 431 KB)
- Table S6 - Genotypic classes sizes at SSR markers along chromosome 4, and chi-squared (*χ*2) statistic for goodness-of-fit with Mendelian 1:2:1 expectations, in the cross Fd50 x WC366. (PDF, 431 KB)
- File S1 - Genotypic and phenotypic data for RHB QTL analysis for the two populations (.zip, 13 KB)
- Table S1 - LOD score, Additivity, Dominance and R2 values of Composite Interval Mapping analysis on rice chromosome 4, for RHBV symptoms in the Fd50 x WC366 cross (.xlsx, 11 KB)
- Table S2 - LOD score, Additivity, Dominance and R2 values of Composite Interval Mapping analysis on rice chromosome 4, for RHBV symptoms in the Fd2000 x WC366 cross (.xlsx, 11 KB)
- Table S3 - LOD score, Additivity, Dominance and R2 values of Composite Interval Mapping analysis on rice chromosome 5, for mechanical damage symptoms caused by *T. orizicolus* in the Fd2000 x WC366 cross (.xlsx, 12 KB)
- Table S4 - LOD score, Additivity, Dominance and R2 values of Composite Interval Mapping analysis on rice chromosome 7, for mechanical damage symptoms caused by *T. orizicolus* in the Fd50 x WC366 cross (.xlsx, 15 KB)
