# Supplementary material for: Major QTLs Control Resistance to Rice Hoja Blanca Virus and Its Vector Tagosodes orizicolus
Source: G3 (Bethesda). 2013 Nov 15;4(1):133–42. doi: 10.1534/g3.113.009373 (PMC3887529; doi:10.1534/g3.113.009373)
Supplement: Supporting Information [file supp_g3.113.009373_009373SI.pdf]

G3·Genes | Genomes | Genetics

Major QTLs control resistance to *Rice hoja blanca virus* and its vector *Tagosodes orizicolus*

Luz E. Romero,[*1] Ivan Lozano,[†1] Andrea Garavito,[*,†] Silvio J. Carabali,[‡] Monica Triana,[†] Natalia Villareal,[†] Luis Reyes,[‡] Myriam C. Duque,[‡] César P. Martinez,[‡] Lee Calvert,[†,‡] and Mathias Lorieux[*,§2]

[*]Rice Genetics and Genomics Laboratory, International Center for Tropical Agriculture (CIAT), A. A. 6713, Cali, Colombia, [§]DIADE Research Unit, Institut de Recherche pour le Développement (IRD), 34394 Montpellier Cedex 5, France, [†]Virology Unit, International Center for Tropical Agriculture (CIAT), A. A. 6713, Cali, Colombia, [‡]Rice Project, International Center for Tropical Agriculture (CIAT), A. A. 6713, Cali, Colombia

[1]These authors contributed equally to the work
[2]Corresponding author: Rice Genetics and Genomics Laboratory, CIAT, A. A. 6713, Cali, Colombia. E-mail: m.lorieux@cgiar.org, mathias.lorieux@ird.fr

DOI: 10.1534/g3.113.009373

File S1

Genotypic and phenotypic data for RHB QTL analysis for the two populations

Available for download as a .zip file at http://www.g3journal.org/lookup/suppl/doi:10.1534/g3.113.009373/-/DC1 and in Qgene v. 4 data format (.qdf) at http://mlorieux.free.fr/Rice_Genomics/Data_QTL-RHB/.

Tables S1-S4
Available for download as Excel files at http://www.g3journal.org/lookup/suppl/doi:10.1534/g3.113.009373/-/DC1

Table S1 LOD score, Additivity, Dominance and R2 values of Composite Interval Mapping analysis on rice chromosome 4, for RHBV symptoms in the Fd50 x WC366 cross

Table S2 LOD score, Additivity, Dominance and R2 values of Composite Interval Mapping analysis on rice chromosome 4, for RHBV symptoms in the Fd2000 x WC366 cross

Table S3 LOD score, Additivity, Dominance and R2 values of Composite Interval Mapping analysis on rice chromosome 5, for mechanical damage symptoms caused by *T. orizicolus* in the Fd2000 x WC366 cross

Table S4 LOD score, Additivity, Dominance and R2 values of Composite Interval Mapping analysis on rice chromosome 7, for mechanical damage symptoms caused by *T. orizicolus* in the Fd50 x WC366 cross

Table S5 Genotypic classes sizes at SSR markers along chromosome 4, and chi-squared (χ^2) statistic for goodness-of-fit with Mendelian 1:2:1 expectations, in the cross Fd2000 x WC366. Htz: Heterozygote. *p*: probability associated to the χ^2 statistic

**Marker**	**Position (cM)**	**Fd2000**	**WC366**	**Htz**	**Sum**	**χ^2 (1:2:1)**	***p***
RM335	0.0	45	45	111	201	2.19	0.33387
RM518	7.2	51	42	125	218	5.44	0.06586
RM16368	9.4	49	45	124	218	4.28	0.11794
RM6770	10.3	49	45	124	218	4.28	0.11794
RM16393	12.9	41	46	131	218	9.11	0.01051
GRCR4	13.6	42	44	132	218	9.74	0.00766
RM16413	16.2	43	36	139	218	16.96	0.00021
RM16416	16.9	45	37	136	218	13.96	0.00093
RM627	27.8	65	31	122	218	13.71	0.00106
RM16459	43.6	71	44	103	218	7.35	0.02537
RM1305	51.3	53	53	111	217	0.12	0.94402
RM6659	52.7	55	52	110	217	0.12	0.93968

Table S6 Genotypic classes sizes at SSR markers along chromosome 4, and chi-squared (χ^2) statistic for goodness-of-fit with Mendelian 1:2:1 expectations, in the cross Fd50 x WC366. Htz: Heterozygote. *p*: probability associated to the χ^2 statistic

Marker	Position (cM)	Fd50	WC366	Htz	Sum	χ^2 (1:2:1)	*p*
RM335	0.0	61	75	146	282	1.74	0.41797
RM518	4.7	53	69	149	271	4.58	0.10130
RM16368	6.2	59	74	156	289	3.39	0.18382
RM6770	8.2	52	72	157	281	6.72	0.03469
RM16393	9.7	44	70	172	286	16.49	0.00026
GRCR4	12.1	47	60	184	291	21.54	0.00002
RM16413	14.1	43	43	169	255	27.02	0.00000
RM16416	16.4	55	46	179	280	22.31	0.00001
RM627	17.9	61	47	179	287	18.93	0.00008
RM6487	31.3	44	62	185	291	23.67	0.00001
RM16459	41.8	46	70	165	281	12.64	0.00180
RM6659	45.7	52	66	159	277	7.48	0.02371
RM401	57.5	58	83	139	280	4.48	0.10653
RM7181	81.0	45	59	157	261	12.26	0.00217
