# Supplementary material for: Major QTLs Control Resistance to Rice Hoja Blanca Virus and Its Vector Tagosodes orizicolus
Source: G3 (Bethesda). 2013 Nov 15;4(1):133–42. doi: 10.1534/g3.113.009373 (PMC3887529; doi:10.1534/g3.113.009373)
Supplement: Supporting Information [file supp_g3.113.009373_TableS5.pdf]

Table S5 Genotypic classes sizes at SSR markers along chromosome 4, and chi-squared (χ^2) statistic for goodness-of-fit with Mendelian 1:2:1 expectations, in the cross Fd2000 x WC366. Htz: Heterozygote. *p*: probability associated to the χ^2 statistic

**Marker**	**Position (cM)**	**Fd2000**	**WC366**	**Htz**	**Sum**	χ^2 **(1:2:1)**	***p***
RM335	0.0	45	45	111	201	2.19	0.33387
RM518	7.2	51	42	125	218	5.44	0.06586
RM16368	9.4	49	45	124	218	4.28	0.11794
RM6770	10.3	49	45	124	218	4.28	0.11794
RM16393	12.9	41	46	131	218	9.11	0.01051
GRCR4	13.6	42	44	132	218	9.74	0.00766
RM16413	16.2	43	36	139	218	16.96	0.00021
RM16416	16.9	45	37	136	218	13.96	0.00093
RM627	27.8	65	31	122	218	13.71	0.00106
RM16459	43.6	71	44	103	218	7.35	0.02537
RM1305	51.3	53	53	111	217	0.12	0.94402
RM6659	52.7	55	52	110	217	0.12	0.93968
