# Supplementary material for: Major QTLs Control Resistance to Rice Hoja Blanca Virus and Its Vector Tagosodes orizicolus
Source: G3 (Bethesda). 2013 Nov 15;4(1):133–42. doi: 10.1534/g3.113.009373 (PMC3887529; doi:10.1534/g3.113.009373)
Supplement: Supporting Information [file supp_g3.113.009373_TableS6.pdf]

Table S6 Genotypic classes sizes at SSR markers along chromosome 4, and chi-squared (χ^2) statistic for goodness-of-fit with Mendelian 1:2:1 expectations, in the cross Fd50 x WC366. Htz: Heterozygote. *p*: probability associated to the χ^2 statistic

**Marker**	**Position (cM)**	**Fd50**	**WC366**	**Htz**	**Sum**	χ^2 **(1:2:1)**	***p***
RM335	0.0	61	75	146	282	1.74	0.41797
RM518	4.7	53	69	149	271	4.58	0.10130
RM16368	6.2	59	74	156	289	3.39	0.18382
RM6770	8.2	52	72	157	281	6.72	0.03469
RM16393	9.7	44	70	172	286	16.49	0.00026
GRCR4	12.1	47	60	184	291	21.54	0.00002
RM16413	14.1	43	43	169	255	27.02	0.00000
RM16416	16.4	55	46	179	280	22.31	0.00001
RM627	17.9	61	47	179	287	18.93	0.00008
RM6487	31.3	44	62	185	291	23.67	0.00001
RM16459	41.8	46	70	165	281	12.64	0.00180
RM6659	45.7	52	66	159	277	7.48	0.02371
RM401	57.5	58	83	139	280	4.48	0.10653
RM7181	81.0	45	59	157	261	12.26	0.00217
